# Recent trends in real estate research: a comparison of recent working papers and publications using machine learning algorithms

**DOI:** 10.1007/s11573-020-01005-w

**Published:** 2020-09-05

**Authors:** Wolfgang Breuer, Bertram I. Steininger

**Affiliations:** 1 Department of Finance, RWTH Aachen University, Templergraben 64, 52056 Aachen, Germany; 2 KTH Royal Institute of Technology, Real Estate Economics and Finance, Teknikringen 10 B, 100 44 Stockholm, Sweden

**Keywords:** R30, C45, C80

## The economic relevance of the real estate sector und its recent dynamics

In most countries, the real estate sector plays a dominant role—as measured by volume, share of the economy, and workforce; but its turnover ratio and therefore its ability to generate profit for traditional banks or transaction and consultancy companies is lower than it is for the stock or bond market. For example, in the US, the total value of the real estate market amounted to about $46.4 trillion in 2018 (Savills 2017, 2019) which is around half of American households’ overall net wealth of $98.2 trillion in 2018 (Credit Suisse 2018) and about 2.3 times the US GDP of 2018; around 4.5% of the US workforce^1^ works in the construction sector in 2018 (BLS 2019). Similar figures can be obtained for Germany with a total value of the real estate market of about $8.3 trillion in 2018 (Savills 2017, 2019) which is more than half of the households’ overall net wealth of $14.5 trillion in 2018 (Credit Suisse 2018) and about 2.1 times Germany’s GDP of 2018; around 6.8% of Germany’s workforce^2^ works in the construction and real estate sector in 2018 (Statistisches Bundesamt 2019). However, not only the sheer volume and workforce of the real estate market contributes to its economic relevance, but also its interconnection via financial instruments like mortgages and asset-backed securities with the capital markets, as has been revealed during the subprime crisis from 2007 on. This crisis started in the US real estate sector, but also affected the capital markets and eventually the real economy of many other countries around the world leading to a worldwide recession in 2008/09. The higher utilization of derivatives on financial real estate products used to increase the turnover of the business with real estate and amplified this crisis.

Besides this apparently already high general economic importance of the real estate sector, there are several reasons why to expect rather a highly dynamic development in this segment even for the near future. According to personal interviews and surveys with 905 influential real estate experts (PWC and ULI 2020) and market analyses of real estate consultant firms (e.g., JLL 2020; Savills 2020), there are the following megatrends at work which will change business in the real estate industry in the long term and to a large extent: (1) Urban expansion creates further megacities, (2) regulatory changes increase housing affordability in the cities, (3) demographic change affects demand, (4) developers must use new technology to be fast and smart, (5) labor and material costs for construction will continue to rise, and (6) sustainability considerations change the design for buildings. Apparently, not all of these impact factors are also relevant for the special case of Germany. However, certainly demographic change is. In addition, as pointed out by Cajias et al. (2020b), Germany has experienced a long-lasting economic expansion for the last ten years while interest rates – due to the politics of quantitative easing applied by the European Central Bank – are at historically low levels. Induced by these favorable economic conditions in Germany and the general geopolitical situation, high numbers of migrants have come to Germany especially since 2015. Moreover, at least partly as a consequence of an aging population, urbanization, and a growing tendency to single-households, the number of households has been increased. All these aspects have led to a booming demand for housing in the major cities in Germany. Similar trends can be observed for other major European countries (Battistini et al. 2018; Pittini et al. 2019); Germany has a total housing stock of 41.97 million units with 305,659 housing starts in 2017; for France and the UK, the corresponding figures are 35.80 million units with 430,000 housing starts, and 28.74 million units with 193,390 housing starts, respectively (Pittini et al. 2019).

## Real estate conferences and the real estate sector in Germany

Against this background, there is no doubt that a closer look at the German real estate market may be of special interest. However, “business is local” and this insight applies to the real estate sector in a particular way. Therefore, the development of prices and liquidity on the German housing market is not homogenous throughout the country, but rather varying from region to region (Cajias et al. 2020b). In fact, increasing prices can primarily be observed in the most populous cities and their surroundings as well as certain economically strong regions while other parts of Germany, e.g. in the east, may even face decreasing housing prices. As a consequence, a regional analysis of the German housing market is necessary. Three out of the four articles of this special issue address this topic, while the fourth is related to the field of real estate finance.

**Fig. 1:**
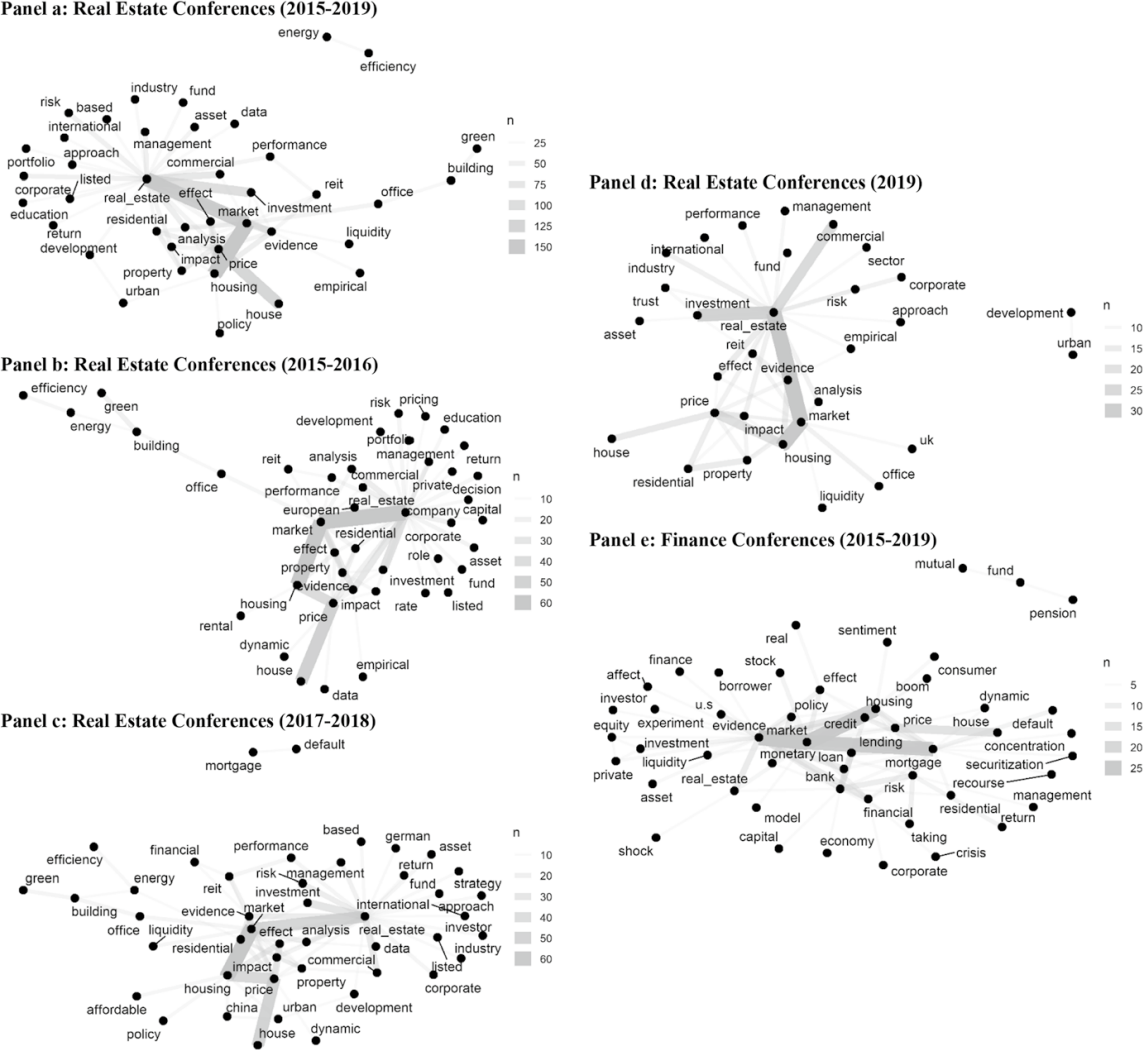
Word Pairs in Titles. This figure shows the most common word pairs in the titles of working papers presented at real estate conferences (annual meeting of the American Real Estate Society, international conference of the American Real Estate and Urban Economics Association, annual meeting of the European Real Estate Society) in Panels **a**–**d** and of real estate related working papers at finance conferences in Panel **e** between 2015 and 2019. For a description of the finance conferences, see Table 1. The number of co-occurrence (*n*) is indicated by the thickness of the connection line; the most common pairs are located in the center

To put these topics in relation to the working papers currently presented at real estate conferences, we analyze the titles of the last papers discussed at annual meetings of the American Real Estate Society and the European Real Estate Society, as well as at the international conference of the American Real Estate and Urban Economics Association between 2015 and 2019. Since we do not have access to the abstracts of all working papers, we rely on the titles to discover the most common topics currently researched. By using a machine learning method, we extract which words commonly occur together in the titles and plot network figures of the words for their co-occurrences (see Fig. 1). The plot of these co-occurring words identifies the relationships better than a table. To focus on the keywords, we ignore stop words such as ‘and’ or ‘or’ and swapped all plural words to the singular. For the entire time period (Panel a), the word family is clustered around *real estate* in the center. The terms *market*, *house*, and *housing* build a second word family with fewer ties to other words than *real estate*. Nevertheless, the network has a clear center and not various clusters with word families. Surprisingly, the word *mortgage* is not in the focus of research. The pair *energy* and *efficiency* is isolated and the pair *green* and *building* is connected via *office* to the center. In Panels b, c, d, we analyze a shorter period of time and find that the pairs *energy* and *efficiency* as well as *green* and *building* were connected to the center for the years 2015–2016 (Panel b), whereas *mortgage* and *default* were isolated in the years 2017–2018 (Panel c). For the recent year 2019 (Panel d), the map shows a similar network as for the entire sample, but we see that the *UK* and *market* may be more in the focus due to the Brexit.

Taking now a closer look at the articles of this special issue that aim at the analysis of the German real estate market we indeed find that none of them is concerned with mortgage issues, but all of them address general characteristics and developments of the German real estate market.

According to the study “Transformation of the real estate and construction industry: empirical findings from Germany” by *Andreas Pfnür* and *Benjamin Wagner*, the specific drivers of structural change on the German real estate market are more important than the general mega-trends mentioned above (Pfnür and Wagner 2020). Based on a comprehensive survey, the following main determinants of the transformation process in the German real estate industry are identified: Massive changes in space requirements and the way space is provided induce occupiers to search for holistic and flexible solutions to fight increasing uncertainties regarding their requirements for space. As a consequence, property developers concentrate on their development activities more on the occupiers. While investors are also recognizing this rising relevance of occupiers, for the time being, they often refrain from reacting to this in an adequate way. Instead of questioning their existing business models in a general way, service providers try to optimize already existing processes and activities. Summarizing, up to now, the players in the real estate industry have failed to satisfy the altered needs of occupiers—an issue which may constitute a threat to the transformation process in Germany in general.

*Marcelo Cajias*, *Philipp Freudenreich*, *Anna Freudenreich*, and *Wolfgang Schaefers* are looking at “Liquidity and prices: a cluster analysis of the German residential real estate market”, thus investigating also in a differentiated way regions instead of the country as a whole (Cajias et al. 2020b). Based on a dataset that comprises in total more than 4.5 million observations in 380 German regions from the beginning of 2013 to the end of 2018, the authors first build on a regional basis quality- and spatial-adjusted price indices as well as a liquidity index for the most important German residential investment and rental markets. A cluster analysis leads to the result that the optimal number of clusters is two for price as well as liquidity. For both price and liquidity on the investment and rental market, the first cluster is characterized by higher growth rates with respect to population, working population, and real GDP leading to a more pronounced demand for space. Moreover, this first cluster generally exhibits lower unemployment rates and higher disposable income. As another consequence of this study, it seems that a large part of the German population has turned into professional real estate investors, because the regions belonging to the first cluster appear to be chosen with the help of a very sophisticated market analysis in order to identify those areas with the best fundamental data.

In their article entitled “Exploring the determinants of real estate liquidity from an alternative perspective: censored quantile regression in real estate research”, *Marcelo Cajias*, *Philipp Freudenreich*, and *Anna Heller* present the first study to investigate the time on market for rental dwellings (i.e. the inverse of their liquidity) by applying a censored quantile regression (CQR) in real estate research (Cajias et al. 2020a). By this approach, the variation of time on market is explained as a function of dwelling features and other spatial and socioeconomic characteristics. In particular, CQRs make it possible to model any quantile of the distribution of the dependent variable. The underlying dataset consists of 482,196 observations on the rental market of Germany’s seven largest cities between the beginning of 2013 and the end of 2017. The relevance of explanatory variables for time on market differs across these cities and between time on market quantiles in the cities under consideration highlighting the relevance of this special empirical approach and one more time of a detailed market assessment. Against this background, landlords of dwellings should be better able to infer how fast they can let them or what measures could be taken to increase their marketability. Moreover, the conclusions of this article hint at the problems connected with nationwide or statewide policy measures instead of addressing particular regions, cities, or neighborhoods.

## Real estate finance as a special subset of the real estate literature

As a second pillar of our empirical analysis, we want to get a better feeling of the relevance of real estate related topics in finance. Therefore, we conduct an examination of the most relevant finance conferences (annual meetings of the American Finance Association, the Western Finance Association, the European Finance Association, the Financial Management Association (US Meeting), and the European Financial Management Association) for the years 2015 to (May) 2020 and the most important finance journals (Journal of Finance, Journal of Financial Economics, Review of Financial Studies, Review of Finance, Journal of Financial and Quantitative Analysis, Journal of Banking and Finance, and Journal of Corporate Finance). We searched for articles and papers with keywords containing at least one out of the following list: agricultural, housing, land, mortgage, real estate, residential, REIT, rural, spatial, and urban. The overall number of such real estate related finance papers and articles are described by Table 1. About 5.6% of all papers presented at the most relevant finance conferences are related to real estate issues. While this figure is rather stable over time, there are marked differences across conferences with the highest shares of real estate topics for the American Finance Association and the Western Finance Association. The smallest numbers of such papers are presented at the annual meetings of the European Financial Management Association. A similar average portion of about 6.1% results when we look at the journal articles. Once again, the overall average does not exhibit much intertemporal variation. Figures are remarkably high for the Journal of Financial and Quantitative Analysis (about 19%) whereas (with the exception of the Journal of Finance, about 8%) for the other journals, they are mostly in a range of 4–6%.

**Table 1:** Real estate related finance articles and papers

	2015	2016	2017	2018	2019	May 2020
Panel a: Conferences						
American Finance Association	29	32	28	31	31	24
	14.7%	12.7%	11.9%	12.6%	12.9%	10.0%
Western Finance Association	19	19	22	26	17	20
	13.1%	13.1%	15.3%	18.1%	11.8%	13.7%
European Finance Association	20	22	11	8	3	
	8.3%	9.1%	5.0%	3.3%	1.2%	
Financial Management Association (US Meeting)	17	24	17	25	22	
	2.3%	2.8%	2.2%	3.3%	3.2%	
European Financial Management Association	4	3	4	7	1	
	1.4%	1.0%	1.2%	2.3%	0.4%	
Total (Conferences)	89	100	82	97	74	44
	5.5%	5.6%	4.8%	5.7%	4.7%	11.4%
Panel b: Journals						
Journal of Finance	7	3	8	4	3	4
	9.6%	4.1%	12.9%	6.2%	4.3%	10.5%
Journal of Financial Economics	4	5	4	4	7	5
	3.3%	4.1%	3.3%	3.5%	5.1%	5.8%
Review of Financial Studies	6	6	6	10	7	4
	6.9%	6.5%	5.4%	8.3%	5.7%	4.9%
Review of Finance	3	2	2	4	2	2
	5.2%	2.9%	2.8%	6.5%	6.1%	10.5%
Journal of Financial and Quantitative Analysis	12	12	18	15	8	11
	22.2%	17.6%	19.8%	17.9%	9.9%	27.5%
Journal of Banking and Finance	12	7	6	10	4	6
	4.0%	4.1%	3.5%	4.2%	2.1%	4.0%
Journal of Corporate Finance	6	10	9	3	5	5
	5.4%	8.2%	5.7%	2.2%	4.8%	3.8%
Total (Journals)	50	45	53	50	36	37
	6.2%	6.3%	6.7%	6.1%	4.8%	6.8%

This table shows the absolute and relative number of real estate related finance working papers in relation to all papers for various conferences (Panel **a**) and journals (Panel **b**) for the years 2015 to (May) 2020. The respective key words to be incorporated in this list are: agricultural, housing, land, mortgage, real estate, residential, REIT, rural, spatial, and urban

Not very surprisingly, results with respect to the frequency of our keywords are rather unambiguous as well. When first looking at the conference papers, from all references to this keyword list, about 83% belong to the following terms: *real estate*, *mortgage*, and *housing*. If papers are weighted according to their respective downloads in the Social Science Research Network, almost the same result applies: About 81% of all downloads refer to papers which contain at least one of the three terms *real estate*, *mortgage*, and *housing*. When turning to the results for the finance journals, the ranking slightly differs, but one more time, the terms *mortgage*, *real estate*, and *housing* perform best. However, only about 65% of all real estate related references stem from these keywords. With articles being citation-weighted, this figure decreases slightly to 61%. The next two most frequent keywords are *residential* and *rural* with an overall share of about 14 to 18%.

Apparently, real estate-related finance papers are typically addressing issues in the sphere of mortgages. Comparing the results with a pair word network as in Fig. 1, we are able to identify a center around *evidence* which is closely and strong connected to *market*, *housing*, *credit*, and *mortgage*. The term *real estate* is not in the focus of finance related conferences and the terms *mutual pension fund* is isolated (see Panel e).

**Fig. 2:**
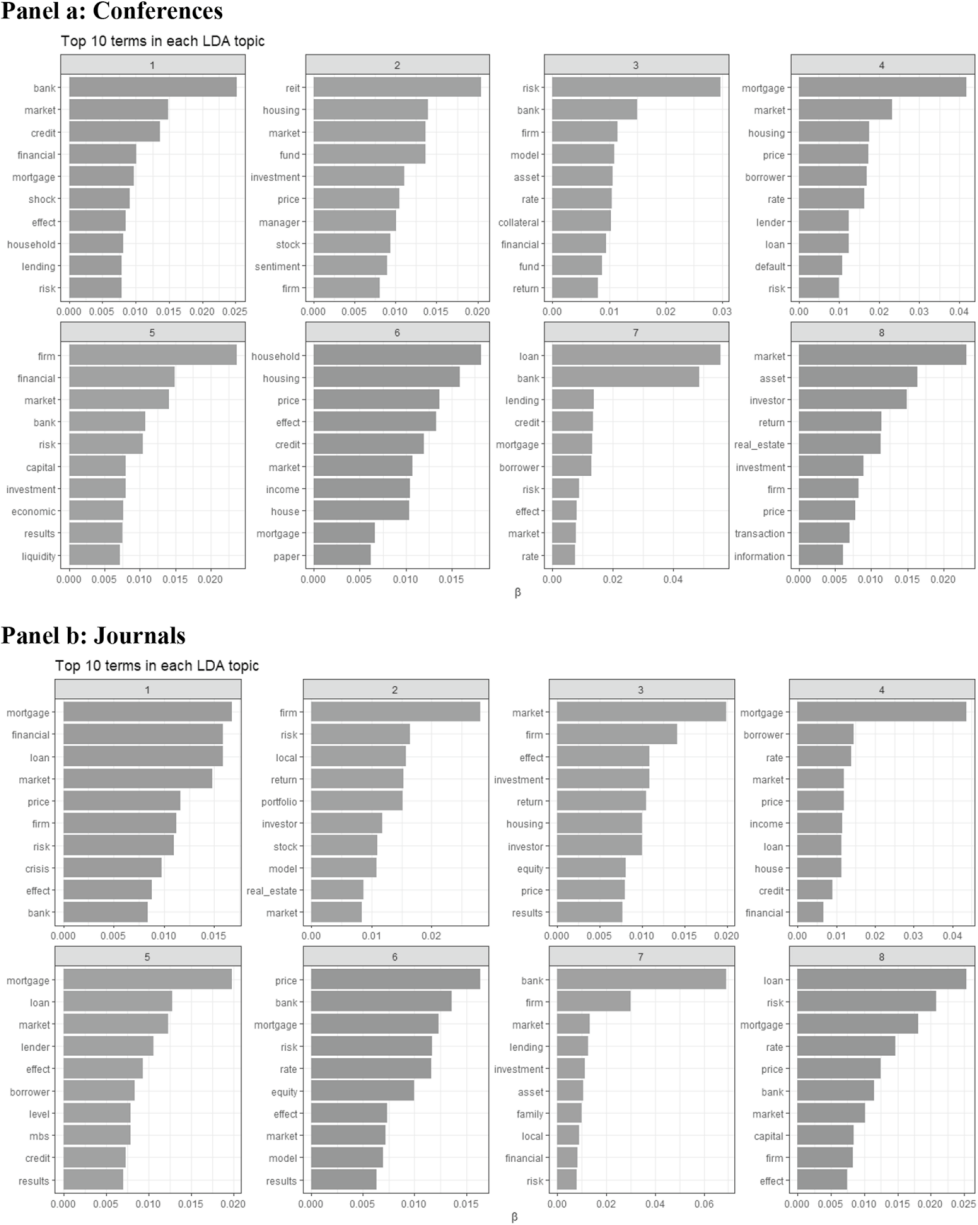
Probability of a word belonging to a topic. This figure shows the top 10 words for each of the 8 topics found by the LDA (Latent Dirichlet Allocation) in the abstracts of the finance conferences (Panel **a**) and finance journals (Panel **b**) for papers with real estate related subjects. For a description of the conferences and journals, see Table 1. Each word is connected with a probability (*β*) of that word belonging to that topic

**Fig. 3:**
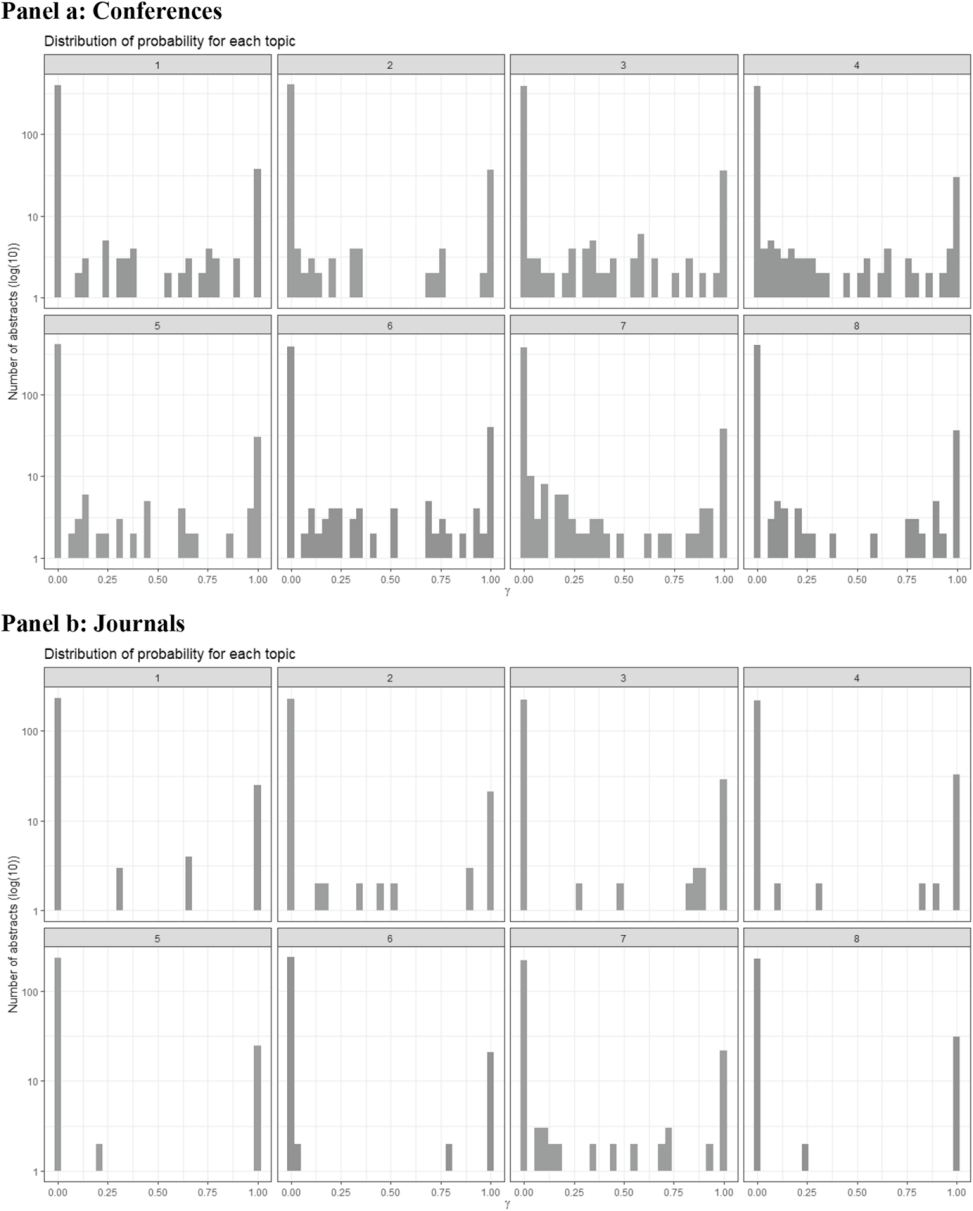
Probability of an abstract belonging to a topic. This figure shows the probability (*γ*) that a real estate related abstract belongs to specific topic. The y-axis counts the number of abstracts and is log(10)-scaled. Panel **a** depicts the finance conferences and Panel **b** the finance journals; see Table 1 for a description of the conferences and journals

To take a somewhat more differentiated and state-of-the-art view, we additionally performed a machine learning method to identify the common “topics” in the abstracts of the conference papers and publications under consideration (see Figs. 2, 3). To find the topics, we apply the text mining approach “Latent Dirichlet Allocation” (LDA) (Blei et al. 2003); this unsupervised machine learning method classifies each abstract as a mixture of topics and each topic as a mixture of words. This approach mimics the natural language processing for dividing documents into natural groups without any pre-specified topics. The mathematical approach is conceptually similar to a factor analysis, where a large number of variables is reduced to fewer factors. The same idea applies to LDA where the large dimensionality of linguistic data is reduced from words to topics (Dyer et al. 2017). In line with the word pairs of Fig. 1, we use word co-occurrences within documents (in our case abstracts), but topics are defined as a collection of words and each word is linked with a probability of belonging to a topic. Thus, LDA connects documents with probability distributions belonging to topics, so that one document can contain several topics. Based on the number of observations, we set the number of topics to eight. For an overview how to apply text mining with the programming language R, see Silge and Robinson (2016).

In Fig. 2, we show the probability (*β*) of the top 10 words for each topic belonging to that topic—conferences are in Panel a and journals in Panel b. Due to space considerations, we refrain from a detailed analysis of the results. For conferences and journals, in six or seven, respectively, out of eight topics we find at least one of our most important keywords *mortgage*, *real estate*, and *housing*, and apparently most clusters are focusing on financing problems with respect to real estate properties for private households and firms as well as the risks connected with such transactions. Moreover, in this regard, the role of mortgages as collateral as well as the role of banks and mortgage-backed securities (MBS) in secondary-market transactions are discussed. Even crisis aspects are addressed. Additionally, though of somewhat minor importance, there is some reference to real estate transactions from an investment point of view e.g. with more emphasis on return issues (see especially topics 2 and 8 for conferences and topics 2 and 3 for journals). Overall, clustering results for our finance conferences and finance journals are rather similar, so that at least for the last five years, there does not seem to be a special development in real estate topics anticipated by finance conferences in comparison to the subjects currently addressed in finance journals. Thus, we will see similar topics for the next year(s), since conference papers have an average lead time of at least 1 to 2 years before they are published.

However, the “unique allocation” of the topics is different between the finance conferences and finance journals as shown in Fig. 3 with the probability (*γ*) that a given abstract belongs to a given topic. In Panel b (journals) we see with topics 5 and 8 that the majority of the articles does not cover this topic (0%), whereas most of the remaining abstracts cover this topic for 100%—it is an either-or topic. The other articles have a similar U-shaped distribution among their corresponding topics. Contrary for Panel a (conferences), the probability that a given abstract belongs to a given topic is here more dispersed. For example, topics 4 and 7 express a bulk of occurrences with low probabilities (left-hand side of the axis).

Thus, conference papers have—currently or in general—a more diluted allocation to the topics than articles, whose abstracts have a clearer distinction among the topics and are higher discriminated as belonging to or not to a topic. Maybe, this is a consequence of the journals’ reviewing process with referees demanding a clearly defined focus of the submitted papers.

Against the background of our results so far, it does not come as a surprise that *Florian Manz*, *Birgit Müller*, and *Dirk Schiereck* are analyzing the special role of real estate collateral in their article “The pricing of European non-performing real estate loan portfolios: evidence on stock market evaluation of complex asset sales” (Manz et al. 2020). Their study is built upon a unique transaction database of 476 non-performing loan deals during the years 2012 and 2018. This leads to the analysis of the value implications of 317 divesture announcements at 58 listed banks during 2012 and 2018. Two-thirds of all collateral are real estate loans, while the other third consists of consumer loans, corporate loans, and mixed loan pools. Since the latter is a mixture of the other three types, the actual real estate proportion will most probably be even higher. The aim of the study is to reach a better understanding of the characteristics and the functioning of the secondary market for such loans under distress. The findings may also lead to regulatory implications since the European Central Bank deems the issue of non-performing loans to be of high importance.

The results identified by Manz et al. (2020) can be interpreted as robust evidence for a significantly positive stock market reaction at vendor banks following non-performing loan sales with the most important determinants of this positive reaction being a size effect and real estate collateral in these transactions. Apparently, the capital markets consider the sale of real estate non-performing loan portfolios to be relatively more attractive than other kinds of non-performing loan portfolios. The tangibility of real estate loans makes it easier for the contractual partners to determine the value of the loan than, e.g., in the case of consumer loans. In addition, real estate collateral requires specific and interdisciplinary knowledge to be handled adequately leading to high opportunity costs for the creditor. When selling these special non-performing loans with real estate collateral, these demanding tasks are transferred from the vendor bank to the buyer releasing valuable resources which now can be allocated to other fields of operation.

## Outlook: real estate trends and COVID-19

When publishing the call for papers for this special issue, the world had never seen before the Coronavirus SARS-CoV-2 and no corresponding disease called COVID-19. Since then, our daily life has changed dramatically and certainly, the consequences for real estate markets will be tremendous as well. Some effects and implications are clear and already irreversible. For example, the long-existing trend of “death of retail” or “retail apocalypse” (Rasmusson 1999) has been exacerbated; first surveys show that many consumers may permanently change their shopping behavior and stick to shopping online (US: 29%; UK: 42%; Competera 2020). The sales of e-commerce shops will continue to rise and traditional shops have expanded their e-commerce channels in answer to the current situation. This will shift the demand for space of retail shops and shopping centers to storage houses and decentralized distributions centers and consequently their rents will rise. The hotel, leisure, and catering industry—hopefully—may only undergo a short economic drop so that there are lower long-term consequences with respect to real estate issues to be expected in these fields, unless the impact of a reduction in business trips does not become too grave. Hospitals and nursing homes must adjust their hygiene concepts and may need to make technical and structural modifications in their buildings.

Other effects and implications are unclear and further developments are not yet foreseeable. For example, if the trend to work (partly) at home or in decentralized co-working buildings will continue, the necessity for office spaces in the city centers will decrease. This would change the demand for traditional office concepts and commuting patterns and consequently the city and traffic planning.

Even if the implications of the articles in this special issue are not directly affected by the long-term social and economic distortions of the “new” Coronavirus, the consequences of this pandemic for real estate related issues are worthwhile to be examined.

## Footnotes

1 This figure comprises the workforce in the “construction” industry itself without the employees working indirectly for the real estate sector in the “financial”, “governmental” or “other services” sector.

2 This figure comprises the workforce in the “construction” and “real estate and residential housing” sector.

## References

[CR1] Battistini N, Le Roux J, Roma M, Vourdas J (2018) The state of the housing market in the euro area. ECB Econ Bull 7:71–93

[CR2] Blei DM, Ng AY, Jordan MI (2003) Latent dirichlet allocation. J Mach Learn Res 3:993–1022

[CR3] BLS (2019) Employment by major industry sector. https://www.bls.gov/emp/tables/employment-by-major-industry-sector.htm. Accessed 22 June 2020

[CR4] Cajias M, Freudenreich P, Heller A (2020a) Exploring the determinants of real estate liquidity from an alternative perspective: censored quantile regression in real estate research. J Bus Econ. 10.1007/s11573-020-00988-w

[CR5] Cajias M, Freudenreich P, Freudenreich A, Schäfers W (2020b) Liquidity and prices: a cluster analysis of the German residential real estate market. J Bus Econ. 10.1007/s11573-020-00990-2

[CR6] Competera A (2020) COVID-19 shopping behavior: what products would customers rather buy online? https://competera.net/resources/articles/ecommerce-online-shopping-behavior-retail-infographic. Accessed 22 June 2020

[CR7] Credit Suisse (2018) Research Institute: Global Wealth Report 2018. https://www.credit-suisse.com/media/assets/corporate/docs/publications/research-institute/global-wealth-report-2018-en.pdf. Accessed 29 June 2020

[CR8] Dyer T, Lang M, Stice-Lawrence L (2017) The evolution of 10-K textual disclosure: evidence from latent dirichlet allocation. J Account Econ 64(2–3):221–245

[CR9] JLL (2020) Global Market Perspective: Highlights. https://www.us.jll.com/en/trends-and-insights/research/global-market-perspective-may-2020. Accessed 29 June 2020

[CR10] Manz F, Müller B, Schiereck D (2020) The pricing of European non-performing real estate loan portfolios: evidence on stock market evaluation of complex asset sales. J Bus Econ. 10.1007/s11573-020-00983-1

[CR11] Pfnür A, Wagner B (2020) Transformation of the real estate and construction industry: empirical findings from Germany. J Bus Econ. 10.1007/s11573-020-00972-4

[CR12] Pittini A, Julien Dijol J, Turnbull D, Whelan M (2019) The state of housing in the EU 2019: decoding the new housing reality, housing europe, the european federation of public. Cooperative and Social Housing, Brussels

[CR13] PWC and ULI (2020) Emerging trends in real estate: Europe 2019, London. PwC and ULI, London

[CR14] Rasmusson E (1999) The death of retail. Sales Market Manag 151(3):17

[CR15] Savills (2017) The 10 most valuable real estate markets in the world. https://www.savills.com/blog/article/219340/international-property/the-10-most-valuable-real-estate-markets-in-the-world.aspx. Accessed 22 June 2020

[CR16] Savills (2019) Savills Report: How much is the world worth? https://www.smartowner.com/blog/savills-report-how-much-is-the-world-worth. Accessed 29 June 2020

[CR17] Savills (2020) Impacts: the future of global real estate, vol 3, pp 6–13. https://www.savills.com/impacts. Accessed 29 June 2020

[CR18] Silge J, Robinson D (2016) tidytext: Text mining and analysis using tidy data principles in R. J Open Source Softw 1(3):37. 10.21105/joss.00037

[CR19] Statistisches Bundesamt (2019) Erwerbstätige: Deutschland, Jahre, Wirtschaftszweige (WZ2008). Statistisches Bundesamt, Wiesbaden

